# Foreign Medical and Physical Science and Literature

**Published:** 1819-06

**Authors:** 


### FOREIGN MEDICAL AND PHYSICAL SCIENCE AND LITERATURE.

*A Historical and Practical Treatise on Scurvy, as it affects Man and Brute Animals.* By M. Balme, Doctor in Medicine of the Faculty of Montpellier; late Surgeon of the first Class, and ex-Physician to the Army, &c. 8vo. pp. 323. Lyons, 1819.*

FROM the term *Scurvy* having been applied to forms of disease of disputed identity of nature, by various writers, we are at a loss to determine what ideas should be associated with that appellation, without a precise and particular explanation being affixed to it whenever it is employed. Sir John Pringle, Lind, Sir Gilbert Blane, and several other esteemed authors, have considered that the disease which affects persons who have been long at sea, and used the ordinary diet of seamen, or persons confined in close garrisons, deprived of fresh animal food and acescent vegetables, should alone be termed scurvy; whilst several other English, and many of the continental physicians, have thought that a disease of a similar character may occur under various other circumstances, and with the use of different sorts of diet: and to these the term scurvy has, consequently, been also applied. If the latter proposition be correct, this mode of employing the term is evidently conformable to the ordinary method of medical nomenclature; and, whilst the precise etiology of this or these species of disease remains undetermined, it cannot be objected to by arguments deduced from the received principles of nosology. We shall, therefore, use the term with this extensive application, in the following analysis.

M. Balme treats on scurvy as a morbid affection arising under the latter circumstances; and he advances numerous observations, deduced from his own extensive experience, and the writings of other authors, to shew the correctness of the view he has thus taken of the disease. As our intention, in the analysis of the work of Dr. Balme, will be best fulfilled by proceeding from particular to general observations, we shall immediately enter into the consideration of it, and furnish our readers with a progressive abstract of its contents.

The author commences with some general reflections on the nature of the animal functions in health and in disease; the object of which is to prove, that morbid actions are merely modifications of healthy ones: an opinion we have frequently expressed, and

* *Traité Historique et Pratique, du Scorbut, chez l'Homme et les Animaux.* Par M. Balme, Docteur en Médecine de la Faculté de Montpellier, &c. &c. à Lyon, chez Cabin et Co.; et à Paris, chez Gabon.

which we believe Bordeu to have first substantiated. He then adduces some arguments to shew, that a variety, in the nature of external stimulants, is necessary for the preservation of the health of the animal economy.

"These principles," says Dr. Balme, "being granted and applied to scurvy, we shall perhaps perceive, that this cachectic affection is merely the result of a too long continued sameness in the state of the body, (particularly if this permanent regularity applies to several of the most important functions,) and that the disease can only be removed, by submitting the system to the impression of new and various species of stimulants."

In support of the latter ingenious, though hypothetical, opinion, Dr. Balme adduces some observations, to shew that this malady does not arise solely from the use of aliments, the nature of which is essentially bad.

"In general," continues the author, "all the descriptions of scurvy, furnished by the different practitioners who have seen and treated it, would oblige us to admit that a vitiated state of the digestive organs, after the use of a bad species of food, is the most ordinary circumstance, and in a manner, the *sine quâ non* condition of the appearance of this malady; and, at the same time that this disorder of the *primæ viæ* is increased or diminished, according to the greater or less deviation of the state of the skin, or other sympathising organs, from that of health. But is this alteration in the digestive system always the result of the same causes?"

In relation to the above question, the author then refers to observations made by Dodonea,* Hoffmann,† Haller,‡ Millman,§ Joseph Franck,|| Sydenham,¶ Huxham,** Weikard,†† and several other respectable authors, which invalidate such an exclusive opinion; and seem to prove, that any particular regimen, either vegetable or animal, mild or stimulant, will occasionally produce the disease, when persevered in with constant uniformity. Several cases are also related, or referred to, to shew that it is particularly apt to ensue from the use of a diet consisting of a considerable proportion of sugar, with any one species of vegetable. Some of these cases, we must remark, are not described with sufficient accuracy and precision, to satisfy us that they were instances of scurvy; others evinced all the ordinary symptoms of that disease, and were thus termed by the authors by whom they were related. These affections appear also to have been removed

---

* *Med. Obs. exempl. rar.* p. 59.

† *Opera*, t. iii. p. 382.

‡ *Disput.* t. vi. p. 507.

§ *Treatise on Scurvy.*

|| *Bibl. Broun. German.* t. ix. p. 122.

¶ *Opera*, t. ii. p. 99.

** De Haen *Ratio Medendi*, t. iv. p. 166.

†† *Element. di Medic. Pratic.* t. ii. p. 189.

by the use of a different diet from that to which the subjects of them had previously been accustomed.

The author considers that the cases to which he has alluded, prove that scurvy will ensue from a diet of vegetable, as well as from one of animal food.

"But if, in objection to this," he continues, "it is stated, that the Brahmins and our mountaineers are long-lived, although they feed only on vegetables; I would reply, that these individuals, habituated from their infancy to this regimen, correct the defects of it by the pure air which they ordinarily respire, by the light water they drink, by various species of exercises of mind or body, &c."

The influence of variation in the articles of diet, although of the same species, in removing scurvy produced by the above cause, is "annually shewn in the inhabitants of Sologne, who become scorbutic towards the end of September: their faces are then puffed, their strength is depressed, their gums spongy and disposed to bleed, and their skin marked by red blotches.* This cachectic state arises from their eating only bread with a mixture of rye, from July to October, and drinking only water during the same period. But, after this time, they become more active; their countenance, and the colour of their skin, assume their ordinary appearance; and health is at length perfectly restored. This change evidently arises from the alteration they make in their diet at the same time, by using rape-seed as part of their aliment, which is new to them at this season of the year. Such is the case with the poor inhabitants; but the higher class of persons, who despise this food and will not employ it, do not equally participate in the good effects of a change of diet at the same period, and are often obliged to have recourse to medicine, properly speaking, to get relieved from the infirmity we have described."

Dr. Balme next treats of the "*direct or proximate causes of scurvy.*" He here observes, that the appearance of the disease, in consequence of a diet intrinsically of a good quality, can only be explained by admitting that the stomach, from having been accustomed to the long-continued stimulus of the same aliment, ceases at length to be susceptible of its impression, and gradually falls into a state of inertia, which permits, and indeed favours, the action of other enervating causes; such as want of exercise, the distressing passions, &c.† He continues to remark, that disorder of the digestive organs alone, although necessary for the production of scurvy, is not always accompanied with it; in consequence of some other functions, correlative or consentient with those of digestion, making up, in a manner, for the weakness of the latter.

---

* *Soc. royale de Med.* t. i. *Hist.* p. 343; *Mémoires*, p. 68.

† Girtanner stated, as a general proposition, that scurvy arises solely from the abstraction of the habitual and ordinary stimulants of the animal economy. Vide *Gianini, Memor. di Medic.* t. i. p. 145.

It is thus that varied occupation, which entertains and supports muscular action, frequent change of linen, which provokes free and continual cutaneous perspiration,* agreeable passions, which excite the whole system, &c. oppose the occurrence of scurvy.

Dr. Balme adduces several instances, to shew the influence of these adventitious causes in the production of scurvy, both at sea and on land; that is, to shew that a certain species of aliment may, under some circumstances, be used without inconvenience, which, on other occasions, will quickly give rise to scurvy.

"From what has already been stated," he continues, "it may be established, that scurvy is a cachexia, the primitive cause of which is debility of the digestive system, the consequence of a too long continued sameness in the mode of living: the nature of which varies, because of the different regimen that may have been adopted; the character of which is not always constant, since it depends on the disposition of different organs to receive the impressions thence arising; and lastly, the establishment and progress of which always depend on the concourse of several enervating causes."

Dr. Balme then adduces some remarks on the division of scurvy, adopted by some authors, into *acute* and *chronic;* and he very properly considers, that the diseases which have been arranged under the former appellation, are essentially different in their nature from those varieties of morbid affection which the most esteemed authors have agreed to term scurvy. The distinction into *acid* and *alkaline*, he thinks, is founded on physical facts, and dependant on the nature of the aliment employed; the exclusive use of vegetable food tending to produce the former, and that of animal food the latter. He remarks,

"Thus, in the *mammiferi,* we perceive the milk,† and the urine,‡

---

* Derangement in this function has, perhaps, after that of the digestive organs, the greatest influence in the development of scurvy. "When the discharges of perspiration by the skin are copious," says Dr. Alexander Wilson, "the scurvy can never rise to a great height." (*Observations relative to the Influence of Climate;* 8vo. London, 1780.) "It is very rare," Bosquillon remarks, in his notes on Cullen's First Lines, "that scurvy takes place whilst cutaneous transpiration is maintained to a considerable degree." "The suppression of perspiration," according to Colombia, (*Médic. Militaire*, t. v. p. 187,) "is in part the cause of this cachexy. This is so true, that, towards the end of moist winters, the gums of many persons become soft, swelled, and spongy. Scurvy is endemic in the people of the north, who have but little transpiration through the skin; and when scurvy is epidemic, those who do not suffer cutaneous perspiration, ordinarily evince the first symptoms of the disease (Sanctorius)."

† *De Natura et Usu Lactis in diversis Animalibus;* auct. T. Young, M.D. &c. t. viii. § 1 and 6.

‡ Wedekind, *De Naturâ, &c. Morb. primarum Viarum*, p. 10.

tend to alkalescence or acescence, according as the animal which furnishes those fluids is nourished with animal or vegetable substances. Thus, the milk of a woman accustomed to the use of the former conjoined with a moderate proportion of the latter, does not coagulate, not even when it is mingled with acids; whilst that of one who lives almost solely on vegetables, becomes nearly in a state of coagulation at the ordinary temperature of the body,* participating more of the nature of that of an herbivorous animal, with which an infant cannot be fed for any considerable length of time with impunity.† Similar results have been obtained from observations on the milk of bitches, according as their food was vegetable or animal."

The influence of those species of food on the urine, is next considered. The former tends to produce an abundant evacuation of that fluid, and solely causes the presence of saccharine matter in it; the latter induces a copious formation of saline substances.

These observations are adduced to shew the propriety of the latter division of scurvy; which the author considers to be more precise than that of *sea* and *land*, as pointing out the essential, and not the accidental, causes of the disease. The alkaline species is, however, that which commonly occurs at sea, in consequence of the diet to which the subjects of it are there confined; whilst the acid is more frequently seen on land.

This division of scurvy, although not exactly consonant with the author's principles respecting the nature of the disease, is not wholly objectionable, since it points out an important object in the therapeutical measures that should be had recourse to in its treatment.

We shall pass over a long chapter, devoted to the consideration of the *adjuvant causes of scurvy*, since these are so numerous and variable in their degree of influence, as to render a concise exposition of them impracticable: the chief of them will also be so obvious as not to require exposition. In this division of the work, we find the author deviates in several points, in his arguments and conclusions, from the principles with which he commenced this discussion. The following proposition is particularly objectionable:

"Shall we not be authorized," says Dr. Balme, "from what has been stated, to consider the salivation which often takes place in the treatment of the venereal disease, as a species of scorbutic affection?"

It is very rude pathology, to consider an affection, arising essentially from the presence of a stimulus in the system, and which consists in the consequences of preternatural excitement, as of the same species as one ensuing from the want of excitement, because there may be some slight similarity in some of their symptoms.

This occasional absence of precision of ideas, is the most serious

---

* *Gaz. Salut.* 1786, No. xxxiv. Retz, *Nouv. Inst.* t. ii. p. 424.

† Franck, *Delect. Opus. Med.* t. v. p. 364.

error into which men are apt to fall, on first attempting to generalize an extensive assemblage of previously diffused facts. The above objection will also apply to many of the cases amongst the multitude referred to by Dr. Balme, in other authors: on collating them, we find many of them equally vague from the principles he first advanced, and several of them of a very indeterminate character. There is a difference between choice and extensive erudition, of which all persons do not seem to be fully aware; and the bitter satire of Bordeu* on Haller is too often applicable to authors, who are apt to let their judgment sleep whilst they make a display of their learning.

M. Balme next treats of the modifications scurvy undergoes, according to the age of the patient and various other adventitious circumstances.

In infants, the first symptom is frequently a copious flow of glairy saliva, often of a sanguineous appearance, and of an extremely fetid odour; the maxillary and labial glands become tumefied, and have a tendency to suppurate; the tongue is enlarged; there is great muscular debility; blotches on the skin are not present, but there are sometimes tubercles in the form of ganglions, dispersed in different parts beneath it; the milk-teeth soon become carious, and are succeeded by others, which, although white on their first appearance, soon decay; the mesenteric glands are particularly liable to become affected; the gums become tumefied and readily bleed, and the mouth is covered with an aphthous eruption, somewhat like millet-seed; the body becomes phlegmatic; and a sort of boil often appears about the neck or face, which terminates in a gangrenous ulcer. Scurvy, especially to this extent, is not often witnessed, except in children brought up in hospitals. "The epidemic disease which reigned amongst the children at *La Charité*, at Lyons, in 1796," says the author, "appeared to me to have been really scurvy: it was characterized by general weakness, puffiness of the skin, swelling of the gums, ulcers in the mouth, which became progressively more severe. There was neither fever, oppression, restlessness, delirium, nor diarrhœa, during the first eight days of the disease; fever nor diarrhœa did not ensue, until after the lapse of a fortnight. In this very distressing state, they preserved the proper exercise of their intellectual functions and their appetite, until death; which took place without either convulsions or pains. Their bodies putrefied with great rapidity."

In old persons, the author continues, scurvy chiefly affects the abdominal viscera: it is sometimes accompanied with troublesome diarrhœa, and generally with great debility, of the lower extremi-

---

* "S'il s'agit d'un petit muscle, d'une figure anatomique, d'une discussion curieuse, M. Haller ne s'épargne point; il cite des auteurs avec une abondance qui fait honneur à son érudition; il fait mille pénibles recherches; il instruit son lecteur en le conduisant dans tous les coins de sa bibliothèque; et lorsqu'il s'agit des matières de pathologie, il n'a rien à dire, rien à citer." *Recherches sur les Crises*, § lix.

ties especially. Negroes are generally affected in the same manner: in them, the affection of the bowels often assumes the character of dysentery. Women are more liable to it than men; but it is in them less severe than in the latter. Persons of a lively disposition are not so subject to severe affections of the stomach and other abdominal viscera, when affected with the disease, as those of a melancholy habit. It is more frequent and severe in winter than during any other season of the year, and is especially apt to take place, when favoured by the more essential causes, in cold climates. A moist and cold atmosphere is particularly favourable to its production: under these circumstances, the skin is especially affected with eruptions, and the limbs with œdematous swellings. Dr. Aaskow, to whom the author refers, remarked a difference in the degree in which seamen in the same ship are affected. Those whose cabins were situated in the lower part of the vessel, were always more severely afflicted than those who slept near the upper deck. From what has been stated, and from various other observations, which our limits oblige us to pass over, Dr. Balme concludes, that

"Accidental differences in its character will arise, according as the greater or less debility of the system may dispose the different organs to be particularly influenced by accidental causes. In some scorbutic patients, the liver is especially affected; in others, the spleen, the extremities, the gums, &c. But, notwithstanding this diversity of the symptoms, it is always in the stomach that the malady has commenced; whilst lesion of the other viscera is rather an effect than a cause."

The author then proceeds to shew, that, although scurvy was not until modern times noticed as a distinct disease, it was not unknown to the ancients; and that the affection they describe as similar to scurvy, is mentioned to have arisen under circumstances which accord with those he has considered essential to the production of this malady.

A chapter is then devoted to the consideration of the *symptomatology of scurvy*. This is a valuable part of the work. The general and particular symptoms are examined with much accuracy, and the characteristics of the disease are here determined with precision. Of this we shall take a general, but rapid, view.

"Before we successively pass in review the most striking symptoms of scurvy," says the author, "we shall observe: 1°. That the order in which they appear frequently varies; for, when a person has been debilitated by a fever, or other disorder of long continuance, the gums are almost always first affected, and lassitude accompanies the first period of the disease; whilst, when the patient is deprived of exercise, by a fracture, a contusion, or a wound, it is in these parts where the first symptoms of scurvy appear. 2°. That the symptoms will be evinced more especially in the external parts, in persons who had previously been accustomed to lead an active life; whilst the same affection will more frequently present itself under the form of hypochondriasis, and other nervous affections, in those who are naturally or habitually sedentary, morose, and fatigued by mental languor and uneasiness. 3°. That, in an individual whose nervous system is particularly mobile, and in whom the nervous action consequently predominates over that of the other systems, an improper mode of life, of greater or less continuance, will not produce scurvy characterized by the ordinary symptoms of *humoral* disorder, but rather a nervous affection, varying in extent and severity. And, 4°, that it is in the evening, and during night, that the most marked symptoms of scurvy are exasperated."

A general weakness is ordinarily the first symptom. This debility is chiefly experienced in the muscular system, and not much in the circulatory organs, excepting those of the adipose fluid; the receptacle of which (the cellular tissue) especially participates with debility of the other organs. There exists essentially, as the very principle of scurvy, a want of the natural healthy influence between the different organs, particularly between the abdominal viscera and the cutaneous system: the former being imperfectly excited by improper or ill-elaborated food, communicate their state of disorder to the cutaneous organ; which, in return, has an unfavourable influence on the abdominal viscera, of which it increases, at least secondarily, the morbid state.

The liver is ordinarily implicated in the disease, principally in those of a robust habit of body and who reside in hot climates. When this organ is affected in those living in a cold, moist atmosphere, and in young, lymphatic, and scrofulous subjects, its colour is observed, after death, to be much altered: it is less dark, and is in a manner blanched. The spleen is also frequently affected, and sometimes to such an extent, as to become disorganized. Tumefaction of the gums and fetid breath, are most common in cold and moist climates. These symptoms are often diverted by ulcers on the limbs, and general eruptions on the skin: the latter affections are most common in warm regions. Affection of the gums was rarely witnessed amongst the soldiers attacked with scurvy at Alexandria; and is considered by the author to affect seamen in particular, in consequence of their especial use of saline aliments, spirituous liquors, and the smoking of tobacco. This symptom was particularly prevalent amongst a body of military who were stationed on the summits of the Alps, and who, to obviate some of the inconveniences of a cold and humid atmosphere, almost incessantly masticated and smoked tobacco. Pains in the limbs, especially the lower extremities, are common, and generally early, symptoms of the disease. Pains in the chest with oppressed respiration, are sometimes present; and the author has observed them to be alleviated by the appearance of a more free cutaneous transpiration, or by hæmorrhage from the gums.

The skin, in consequence of its functions being so intimately connected with those of the digestive organs, is the part most commonly affected. Those scorbutic blotches, the author remarks, which not only interest the cellular tissue, but extend also to the muscular substance, are probably owing to the general debility, in which the vascular ramifications participate, and which, preventing the free passage of the blood from the arterial to the venous vessels, occasions it to stagnate and accumulate in the cellular tissue; especially in those parts the least influenced by muscular action. The blood thus stagnant is less subject to the salutary influence of the vital powers than in the ordinary state; it consequently becomes altered in its qualities, and gives rise to sanious ulcers.

The intellectual functions must necessarily be influenced by such a state of the general system, but derangement of them is not usually proportionate to the alteration of the physical strength. They have often remained undisturbed until death, which has taken place without pain, sometimes whilst the patient has attempted to turn himself in bed.

The disorganization of the soft parts, and the acrimonious state of the fluids, affect also the texture of the more solid structures. Thus, as we observe the cicatrization of wounds and ulcers prevented, or destroyed when already effected, in the more confirmed state of scurvy, so it is not rare to witness the destruction of the union of bones that had been fractured, and a preternaturally friable state, or caries, of these parts take place; and they are sometimes found to become fractured by the voluntary muscular actions, apparently in consequence of a commencement of solution of continuity from the want of due nutrition.

The heart and arteries are also much affected in the latter stages of scurvy. The pulse is soft and feeble, and there is a great disposition to palpitation of the heart, and all the symptoms of aneurism of that organ; a disease which, the author observes, is very frequently established in the patients of scurvy. This disorganization of the fibrous and muscular structures is not so evident in that of the nerves. In those cases in which it does, however, occur to much extent, and is accompanied with decomposition of the other tissues, the most severe state of adynamia ever witnessed in the human body is the result.

The above symptoms are dependant on the lesion of the solids; those consequent on the alteration of the different fluids, are now to become the subject of consideration.

The blood in the commencement of scurvy, may have the natural appearance, and be insipid to the taste; although the saliva is at the same time of an acrid quality. But, in the course of the disease, it becomes dark-coloured, its surface greenish, and the serum very acrid; and in the latter stages, this fluid appears dissolved, without albumen, and its integrant parts ill-elaborated, and even confounded. This alteration of the state of the blood, the author considers, is to be attributed to derangement of the organs destined to prepare it, rather than to the ill qualities of the chyle that is mingled with it.

The urine generally varies from the natural state: it is ordinarily irritating in quality, of a deep colour, and small in quantity; it often deposits a brick-coloured sediment; and a saline pellicle is sometimes observed on its surface. These alterations are more evident when the cutaneous transpiration is suppressed. But the state of this fluid varies so much, according to the nature, severity, and period of the disease, that no precise observations respecting it can be advanced: the same remark will also apply to the secretions in general.

We shall pass over the sections of this work which treat of *the incompatibility of scurvy with some diseases; the diseases which bear a marked analogy to scurvy; the complications of scurvy with some other diseases; natural or artificial scurvy in brute animals;* and *the period of the development, and prognosis of scurvy;* as incapable of an analysis compatible with the limits of our Journal. Besides, we consider that attentive and accurate observation, and a little judicious reflection, will supply the medical practitioner with such information as is most necessary on the greater number, and most important, of the subjects above referred to.

We now come to the observations of Dr. Balme on the *medical treatment* of the disease under consideration. This section of the work has given us the most favourable idea of the talents of the author. He here reasons with precision on his own physiological principles, and illustrates his arguments respecting the appropriate therapeutical measures by many valuable facts, deduced from extensive practical observations. We need not, however, dwell much on this part of the subject; since the medical treatment so naturally flows from the theoretical view which he has taken of the nature of the disease.

The basis of the general treatment should be subordinate to measures expressly opposite to the regimen to which the patients may have been previously submitted. There are no medical substances whatever that are calculated to be productive of benefit in all cases; and the abuse of those which physicians have chosen to term expressly *anti-scorbutics*, has often produced scurvy in persons who had the slightest disposition to it. We must not imagine, that the substances which have been efficacious against this affection, always cure it by mingling with and remedying the bad state of the fluids: it is appropriate aliment, measures which improve digestion, a pure and dry air, intellectual amusements, and lastly, an increase of the irritation of the system, which effect the removal of the disease. The stimulant treatment, indicated in scurvy, must vary according to the severity and stage of the disorder. "We must follow the conduct which the Brunonians adopt in the treatment of a disease from direct debility; it will be necessary to commence with the use of gentle stimulants *(præmissis præmittendis)*, and proceed gradually to more active ones. For, in direct proportion to the length of time which an animal body has been deprived of its natural stimulus, or non-natural and habitual, will be the degree of the accumulation of its irritability; and it will become very sensible to the impression of the slightest stimulant which, in one in the state of health would have hardly any effect."

Many cases have been observed to be much alleviated by a natural or artificial hæmorrhage, probably by the change thus induced between the relation of the fluids and solids. When inflammatory congestion is present, it will be right to commence the treatment by abstraction of blood. Purgatives generally have good effects in the first instance, by removing accumulations of viscid matter with which the intestines are loaded, and thus favouring the influence of stimulants. They must, however, be very gentle when there exists any disposition to a dysenteric affection.

After these preparatory measures, and particularly when the disease has been of long continuance, the cinchona will in general be found beneficial; but it must not be employed except when congestions in any of the abdominal viscera do not exist.

When the exclusive use of salted aliment has appeared to have given rise to the disease, fresh vegetables, the juice of oranges, lemons, &c. are generally more efficacious than the Peruvian bark. The alkaline carbonates taken with vegetable acids, are often more beneficial than the pure acids.

It will also be necessary to vary the curative measures from time to time, when the disease is of long continuance; since these agents are apt to lose their influence on the system after a short period, on these occasions.

Moderate exercise; baths, particularly those of the vapour of alcohol, vinegar, &c., are also indicated. In some very severe cases, bark, with opium in large doses, has afforded relief when all the ordinary means had been inefficacious. "May not the good effects which we have obtained from bark and opium," says Dr. Balme, "as well as those which other practitioners have derived from corrosive sublimate, arsenic, &c. depend on the changes which these measures have produced in the states of the digestive system.

"On taking into consideration all that has been stated in this work," continues the author, "and especially the essential cause of scurvy, which we have attributed to a state of asthenia of the digestive organs, consequent on their having been accustomed to the uninterrupted impression of aliments of one and the same species, which, continued for too great a length of time, at length cease to act as stimulants; we repeat, 1°, that this asthenia of the stomach, &c. increased by derangement of the functions of the cutaneous and muscular systems, augments, in return, the lesion of those parts, and *vice versâ;* and 2°, that this asthenia of the primæ viæ may be diminished by a state of vigour and energy, maintained or re-established, of the organs of motion, or of the intellectual faculties, and by a dry state of the atmosphere; and lastly, that, from the frequent absolute inutility of all the antiscorbutic measures hitherto the most exclusively recommended, it appears evident, from precise observations, that neither vegetables nor any other substances, combat scurvy by a specific virtue; but that a change of regimen, favoured as much as possible by a modification in the use of the non-naturals, forms the true basis for the medical treatment of scurvy."

Dr. Balme terminates this work by some *considerations on the qualities, duties, and prerogatives of a Physician;* but these, although judicious and well-arranged, will excite but little interest after the perusal of the Letters of a Gregory.

On revising the observations we have deduced from the work before us, we perceive that they bear a very desultory character, and want that precision of information which it is our desire to convey to our readers, respecting the subjects of our critical labours. This, however, on the present occasion, must be, in a great measure, attributed to the nature of the work whence they were deduced, which is so encumbered with quotations and references, (multitudes of authorities being adduced in support of every proposition), that it is extremely difficult to preserve in view the author's train of arguments on any subject; and, indeed, the perusal of the work is thus rendered so fatiguing, that nothing but the belief that it contains some good and useful principles, could render it at all supportable.

---

*An Essay on the Consequences of the more severe States of Puerperality;* by J. E. Fodéré, M.D. Professor to the Faculty of Medicine at Strasbourg.

*(Concluded from page 446.)*

After having isolated puerperal fever from the various diseases with which it may be complicated, we should enter into a critical examination of the causes to which it has been attributed: perhaps a ray of light may be elicited in the course of this enquiry, that will illustrate the true nature of the malady.

The causes of puerperal fever are numerous, and may be divided into those which existed before parturition; those which were produced during that period; and those which occur after delivery.

*Before Parturition.*—On considering the mode of life of women in general, we perceive that those who are poor continue to pursue during pregnancy, the rudest species of labour; and that those who are rich abandon themselves, when in the same state, to every excess of luxury, to passions, and to venereal pleasures; they continue throughout the whole term, to gorge themselves with food; and their dress, being regulated by fashion, often exerts powerful and injurious compression on the abdomen. This excess in the use of food, is a circumstance of much weight with those who consider the fever of lying-in women as arising from gastric inflammation. They dwell on the disposition of pregnant women to congestions in the bowels; on the depression of spirits and fear which often precedes labour, and which weakens the digestive system; on the vomitings and diarrhœa which frequently occur; on the good effects that have been derived from emetics and purgatives, &c. They add, and pathological anatomy obliges us to consent to it, that, when the intestines have been irritated by acrid matters collected in them, it is not rare to find, after death, traces of actual inflammation, not only in these, but in the whole of the peritoneum and its diverse productions. We are far from denying the eventual power of these causes to produce peritonitis; but everyone will acknowlèdge, that they are too frequent, and puerperal fever too rare, to permit the latter to be considered as their necessary effect. Acrid matter in the bowels may certainly contribute, if not to produce it, to render puerperal fever more severe. It is, however, but rare that the most intense gastric inflammation produces collections of purulent matter in the cavities of the abdomen or thorax; besides which, they are never so formidable, nor so promptly fatal, as puerperal fever. We therefore regard gastric irritation, either as a complication with the disease in question; or, perhaps, as an affection that has often been mistaken for that malady, when evacuants have acted so specifically in its cure.

***During Parturition.***—The largeness of the head of the fœtus; the different obstacles opposed to the facility of its birth; the abuse of instruments, or of the use of the hand during labour; the violent or unappropriate extraction of the placenta; the retention of the placenta, or of coagula of blood, in the uterus; the bandages with which the belly is bound immediately after delivery; the reflux of blood in the viscera contained in the lower part of the abdominal cavity, in proportion as the uterus contracts after delivery, &c.; have been successively accused as the means of producing the most severe consequences of parturition: but we would again say, that the frequency of these should, as causes, induce much more common effects. We daily see, that of several women who have had equally laborious parturition, and who have been delivered by the same process, that some will become affected with puerperal fever or with peritonitis; whilst others will not experience any unpleasant occurrence. We have seen severe symptoms, with tumefaction of the belly, sometimes result from retention of the placenta, or of portions of that organ, or of coagula of blood; but which have been dissipated as soon as the extraneous body has been rejected, even when they have been long previously retained. I do not then fear to affirm, being supported by long experience, that, although laborious parturition, and the manual operations used in such cases, and the various faults otherwise committed, should, and really are sometimes, the causes of the prompt development of puerperal fever; yet, notwithstanding the derangement of the animal economy caused by consensus with the uterus, notwithstanding the Cæsarean operation, &c. this fever is a much less common accident than the existence of these different causes of irritation, however powerful. As to the reflux of blood, it must take place in all cases when an organ is reduced to its primitive form; but such consequences are not witnessed from it. Besides, this mechanical explanation would not shew, why puerperal fever is most frequent in hospitals, and why it sometimes occurs epidemically.

*After Parturition.*—An opinion that was generally spread abroad, and which is still adopted by some physicians possessing but little real knowledge, is that which attributes the inflammation of the peritoneum, the effusions into the cavity of the abdomen and other parts, and the fever of which we are now expressly treating, to metastasis of the milk; or to neglecting to give suck, either from a disinclination to do this, or from the loss of the infant. Others, in imitation of Sydenham, attribute them to suppression of the lochiæ. Many, to refrigeration of the parts about the genitals, induced by the discharges, &c.; and to the little care that some women in this state take of themselves. It is this which has even been adduced, as a reason why women are less frequently attacked with puerperal fever who have had a severe labour, than those in whom that function has been less painful and irksome. We will not deny that, in some individuals, inconvenience has been experienced from the want of the appearance of milk in the breasts; but, on the other hand, if we examine this subject without prejudice, we shall perceive, that the ill consequences of it are not so frequent as generally stated, and that many women escape this secretion, without thence experiencing any inconvenience. Puerperal fever, as I consider it, has been observed after miscarriage, when the breasts did not contain any milk; it has also been witnessed when the mother suckled her infant, and there has not been any deviation from the ordinary course with respect to this function. Reason and experience are equally repugnant to the admission of metastasis of the milk, as a cause of puerperal fever.

As to the lochiæ, the suppression of which might really be a cause of peritonitis, or inflammation of the uterus, this evacuation continues very often during the course of puerperal fever; and they are frequently suppressed, or evacuated only in very small quantity, without ill consequences. This suppression should not then be considered as a constant cause of that malady.

The influence of cold would equally tend to produce inflammation of the uterus and peritoneum; and I regard it as a frequent cause of this accident and of acute dropsy, from which many women perish who leave their beds too early. Nevertheless, after having seen how the women of the lower order of peasantry conduct themselves, as well as girls who are delivered clandestinely, and women who follow armies and keep up with them in their march after delivery, without accidents; I am forced to consider, that the latitude of this cause is infinitely less extensive than a man would believe, who had seen nature only in his study.

These perils, and many others, are traversed by the greater number of women, and the preservative genius of the species leads them through them with safety: some few, however, are stopped in their course. Why this fatal predilection? We must necessarily conclude, that there is in some women a particular disposition to the determination of puerperal fever, with or without peritonitis; and that, in certain cases, it owes its origin to epidemic causes. This disposition, which cannot be always distinguished or foreseen, gives rise to one of the greatest difficulties in the art of healing, especially in women, the diseases of whom are so "*bizarre;*" and it explains the origin of the fatal peritonitis, which has been observed to ensue from the application of a ligature to uterine polypi.

On reflecting seriously on the picture which we have given of puerperality, it will become evident to every unprejudiced person, that this calamitous disposition of lying-in women to the most severe diseases, depends on the excessive exaltation of *sensibility,* to its alteration, to its deviation towards an organ as it were new, which draws to itself at this time all the vital powers. It is well known, that this exaltation in the organs within the department of the great sympathetic nerves, never occurs without successively inducing lesion of the other systems and organic tissues; which takes place with great rapidity, not only under these circumstances, but also in many others unconnected with parturition, when the nerves which I have mentioned suffer primitive lesion. It is notorious also, that, when puerperal fever is simply sporadic, it is those of great mobility of habit who are affected with it; it is imperious, capricious, choleric, women, are its subjects,—states of mind which I have always seen develop themselves, and become exasperated, during the whole course of this disease. Certainly, when I call to mind all the women whom I have seen perish from this fever, I well recollect that the disease commenced in all of them after a fit of anger, although nothing extraordinary had apparently happened to them; whilst other women, who had suffered much, but who were calm and patient, passed this period without disturbance. We should also connect with the idea of puerperal fever, that tendency which lying-in women have to chronic delirium, without any material cause; the frequency of coma and acute delirium, during the whole course of the fever; and the facility with which slight terror will suddenly induce it, in women apparently in the most favourable state. Thus, Franck has seen it instantly produced in lying-in women on the tolling of the passing-bell, who had been alarmed by the remembrance of the death of so many other women in their situation, and which was renewed by this doleful sound. All these considerations well reflected on, all these phenomena, lead me irresistibly to define primitive puerperal fever, isolated from its complications, *a state eminently nervous; an abdominal spasm; during which the functions are interrupted, and the phenomena secondary to parturition (the determination of the fluids towards the breasts, and the flow of the lochiæ,) do not take place, or are ill performed.*

Its being sometimes accompanied with peritoneal inflammation, (which I do not deny,) is of no importance as an objection to the above definition. It is not a rare circumstance to see diseases, where the sensitive and vital system is primitively affected, accompanied with prompt re-action of the circulatory system of the neighbouring parts. It is not repugnant to it, that there is inflammation, or at least something analogous to it, although there be weakness; and examples are often seen, of an organ concentrating in itself all the powers of life, and being affected with inflammation, whilst the other parts of the system bear the character of debility. Typhus itself has often furnished us with opportunities of witnessing this apparent contradiction, either on the opening of bodies, or in the observation of the good effects ensuing from hæmorrhage, whether spontaneous or produced by art. Inflammation may then be present in some instances, and not exist in others, as I have already shewn, without this accident being considered as an essential cause of puerperal fever. In most instances, peritonitis is only an effect; just as we witness inflammations at the termination of malignant fevers, which no person has hitherto thought proper to attribute to inflammation. However, peritonitis and puerperal fever may, in certain cases, arise at the same time, on the occurrence of causes adapted to favour the production of the former; and this distinction of priority of cause and effect appears to me to be of great importance in the treatment of the disease, and in the explanation of the results obtained from contradictory measures.

These explanations are particularly applied to sporadic puerperal fever; as to that which is epidemic, all constitutions are equally attacked by it, after the single circumstance of parturition: and this epidemic character is most frequently observed in hospitals, in unhealthy situations, wherever there is impure air, and in certain constitutions of the atmosphere proper for giving rise to putrid diseases; a consideration which should again caution us against the exclusive idea, adopted by men for whom I have the greatest respect, of the existence of inflammation of the peritoneum in puerperal fever, being an essential occurrence.

I acknowledge, however, that this purely nervous character is sometimes hardly to be distinguished solely and isolated; that most frequently it is observed complicated with an inflammatory diathesis, at least in the commencement, or with gastric inflammation; and, in epidemic cases, with putrid or adynamic fever, or with symptoms produced by lesion of some organ which was in a diseased state before parturition: but it will always be useful to consider, that it constitutes the foundation of the disease, and that this must not be submitted, in all cases, to the same mode of treatment.

Similar to certain miasmata eminently septic, or to the virus of certain serpents which causes death before nature has had time to produce the phenomena of re-action, this nervous state of which I speak, may run into the most complete ataxia (nervous debility). There may be such a concentration of sensibility in the uterus, and such an absence of vital power in the rest of the economy, effected during gestation and parturition, that the slight breath of life which remains may be suddenly extinguished, and before the ordinary symptoms are developed. This is what I

always fear after a severe labour in delicate and sensible women, who are only an extremely mobile walking shadow. In some instances, a slight pain will terminate what more severe and long-continued sufferings had commenced. I have seen a dose of opium, in others, rapidly consume what has remained of a life exhausted by long sufferings and repeated spasms. I have seen a patient, who has been sickly and languishing during pregnancy, become suddenly extinct, after having had a regular labour without hæmorrhage. What is still remarkable in these instances of sudden death of lying-in women, is, that the infants are almost always born dead. I cannot find a better comparison with this dreadful catastrophe, than a very acute colic, running through its fatal period within three hours, observed in Spain by Dr. Hurtado, characterized by excruciating pain in a fixed point, happening suddenly to persons who had suffered severe moral depression, accompanied with frequent, vibrative, and contracted, pulse; cold sweats; rapid change of the countenance; and lastly, followed by gangrene of the intestines. The theory which Dr. Hurtado formed of this disease, enabled him to save many of its subsequent patients.

The *nervous fever* of lying-in women, or puerperal fever, is insidious; and the treatment of it is so difficult, that the most sure is certainly to use all our efforts to prevent it. It has become the fashion to contemn everything that was formerly considered to be temporising and prudent, but our fathers, it appears to me, were, on the subject under consideration, much more wise than we are; and sentiment guided by experience, led them to take many precautions in the management of women before and after parturition, which are now, perhaps improperly, considered to have arisen from prejudices. I cannot enter into an examination of all these precautions, which may be found in all treatises on the subject; I shall content myself with an enumeration of the principal ones; for example;

1°. Those which relate to the state of pregnancy. They had been accustomed, from time immemorial, to have recourse to blood-letting on the approach of the period of delivery, if the woman was plethoric; and this custom should be preserved as a good one: the abuse of it is only to be reproved. To remedy the effects of the pressure which the uterus exerts on the surrounding parts, they advised a diminution of the quantity of animal food and fermented and stimulating drinks, towards the termination of pregnancy; to keep the bowels lax with mild aperients, or with glysters; and the use of exercise in the open air. The choice of regimen should be subordinate to the constitution of the patient. A free and regular state of the bowels cannot be made too general a precept; and, with respect to compression of the abdomen, it should become an object of public prohibition. With regard to exercise, I have already said that the poor use too much: this precept, then, can only concern the rich. Now, although it be right in principle, it will be evident that violent exercise cannot be proper during pregnancy, and that the degree of it must vary according to circumstances. I even believe that repose is necessary for those wretched women to whom exertion is at all times a labour, and who would certainly be much exhausted by any considerable degree of exercise. Happy would it be for us if we could regulate the passions of a pregnant woman! but who can be the master of all the accidents of life, during the long term of nine months?

2°. Relative to delivery. It was not without urgent motives that it has been decreed, that a woman recently delivered should be carried to her bed, and not permitted to walk there; that the bed should be a little warmed; that she should remain in bed at least eight days; that she should not be soon exposed to the air; and that she should not return to domestic occupations before the lapse of forty days. All experienced observers agree, that, of all the causes of disease in lying-in women, the most frequent is that of rising from bed, and exposure to the air, at too early a period. With respect to diet, there have been two opposite opinions: according to one, the use of a restorative regimen has been stated to be necessary, from a persuasion that a lying-in woman should be treated like a person exhausted by fatigue, or long-continued disease: the other has been derived from reflection on their disposition to inflammation; and it has thence been said, that there is no occasion on which a woman should be confined to more restricted regimen. Between these extremes, equally blameable, no other rule can be traced, than that the physician should act according to the strength of the woman, and the condition in which she may be placed. A precept more generally useful, is, that to procure a free state of the bowels (with due precautions) soon after delivery, and to preserve it throughout; especially in women who do not give suck. It may be useful to retain the custom of applying a bandage round the pelvis; but I would forcibly blame an habit of the women of the present day, that of tightly binding-up the abdomen as high as the ribs, in order to prevent them from acquiring a prominent belly; and I am convinced that this conduct is frequently productive of very bad consequences.

Lastly, if, as we have already stated, it is impossible to preserve a pregnant woman from all sorts of disagreeable impressions, so the short term of puerperal confinement does not prevent the probability of the occurrence of the same accidents; and the dreadful consequences which powerful emotions cause during this period, should call forth all our care, prudence, and circumspection, to guard our patient from their influence. We have already repeatedly mentioned, that there are constitutions of the atmosphere, and insalubrious situations, which are very hurtful to puerperal women: if the former cannot be avoided, the second are entirely at our disposition. It is, for example, easy to preserve the greatest propriety in their chambers, by dispelling all unpleasant odours; removing useless persons; preserving a moderate temperature, and frequent renewal of the air, taking care, however, that no current may directly fall on the patient,

It is but rarely that we are called in time to treat puerperal fever in its most simple form. If we are sufficiently fortunate to see the patient at the commencement of the disease, it will perhaps be possible to check the fever suddenly, in combating it during the first shiverings, by warm and slightly-aromatic drinks, and emollient fomentations applied to the abdomen and inferior extremities. If this state continues, we should not hesitate in the first instance, to produce a more general diffusion of the vital powers, by employing cupping-glasses and rubefacients, applied successively to parts extending from the region of the uterus, commencing the train from the pubis, then above the navel, on the chest, and at length between the shoulders and on the arms. Warm barley-water, with elder-flowers infused in it, should be given as drink, to produce perspiration; with anodyne medicines, to procure calmness and sleep, according to circumstances. The strength should be supported by veal or mutton-broth, given every two or three hours; and the bowels should be opened by simple glysters of infusion of chamomile-flowers and honey.

I am persuaded, though I have not yet had sufficient experience to make me very confident, that this mode of treating puerperal fever on its first invasion will, in a great number of cases, be much more salutary than those powerful medicines employed from this or that suspicion, the success of which is subordinate to the hazard of divination. However, I do not deny that the spasm, which I regard as the primitive cause of the pain and tension of the belly, will give rise to more fixed lesions; and besides, we know that complications may soon alter the simplicity of the disease. Authors have hardly ever remarked it except in this form, and it is thence they have deduced their rules for the mode of treatment, which may be distinguished into three methods: the antiphlogistic, purgative, and mixed. We shall take a rapid view of the whole, and indicate the cases to which each may be respectively appropriate, when the disease can no longer be seen in its greatest simplicity.

The first method, recommended exclusively by Leake, who considered this disease as a true inflammation, has in like manner been put in practice in two epidemics of puerperal fever, in 1810 and in 1811, by Ramsbotham, Gordon, and Hey; at London, Aberdeen, and Leeds: the places where the malady raged with greatest violence. Considering this fever as an active inflammatory disease, the practitioners above named judged that its specific treatment consisted in blood-letting to the quantity of from twenty to twenty-four ounces, and even more, according to circumstances, immediately after the invasion of the malady. They afterwards prescribed purgatives in large doses, for which purposes calomel was employed. In addition to this general treatment, leeches were employed to the abdomen, and the consequent hæmorrhage maintained by warm fomentations; with glysters, with or without opium, frequently repeated. They state, from the success with which it was followed, that this method should be boldly employed, and put in practice after the shivering; that, otherwise, it indubitably failed, and only hastened the death of the patients; that, some hours later, palliative measures should only be resorted to, which are all of but little utility. Cinchona, stimulants, and vesicatories, appeared to Mr. Hey to be more injurious than beneficial; (I have also observed the same thing with respect to cinchona, but this does not apply to vesicatories;) opium, camphor, the neutral salts, were of but doubtful efficacy; but digitalis appeared to him to be of incontestible utility.

It is well known that all the methods hitherto employed, having been inefficacious against puerperal fever at the Hôtel-Dieu at Paris, Dr. Doulcet, in 1782, proposed and put in practice the following treatment, which has preserved his name, and which has indeed been efficacious at different epochs. It consists in seizing the instant of the invasion of the disease, to give fifteen grains of ipecacuanha in two equal doses, with an interval of an hour and a half; to repeat the remedy on the following day, although the pain and tension of the belly may be diminished; and to repeat it three or four times, if these symptoms continue; to open the bowels with a potion composed of two ounces of oil of sweet almonds, one ounce of syrup of marsh-mallows, and two grains of kermes-mineral; to continue the use of this potion for eight days, and to give as drink merely infusion of linseed; and lastly, to purge, after this time, with manna and sulphate of potash.

Denman, who has well treated of the fever in question, appears also to have at first considered it merely as arising from gastric irritation; since he speaks of a case that terminated favourably in 1767, which he attributes to emollient glysters and James's powder. However, he soon changed his language, and terminates his essay with remarking, that the disease is originally of a true inflammatory character, affecting one or more parts contained in the abdomen, but promptly contracting a character of putridity more or less marked, according to its intensity, and to the treatment employed during the inflammatory state. We find him, in consequence, have recourse to a mixed method; directing in the first instance one or two blood-lettings, then administering antimonial powder, which was daily repeated, giving in the evening an opiate to calm occasional irritation.

These different measures, and the different mode in which the same practitioner has viewed the disease at different times, indicate alone that this fever, deprived of its primitive simplicity, assumes a different character, according as it is more or less complicated. We have, indeed, frequently seen physicians, deceived by the pain and tumor of the belly and by the idea of peritonitis, lose their patients under the lancet and the application of leeches to the abdomen. The physicians of Aberdeen and Leeds have not stated what directed them in their absolute employment of blood-letting; but I suppose that, in the cases in which it succeeded, the patients were strong and plethoric; and that the disease was of a peculiar inflammatory character, occurring at a season of the year favourable to the production of inflammatory diseases.

I have thought that emetics and purgatives have been particularly useful, to the exclusion of all other remedies, when the signs of inflammation have been wanting; when the patients had committed errors in diet, either during the latter part of pregnancy or after parturition; and when manifest symptoms of irritation of the stomach and bowels assumed a superiority over those of inflammation. One of the most perplexing circumstances, is that in which the disease appears with great debility, real and not merely apparent, (it should be remembered, that plethora and acrid matters in the bowels will produce apparent debility,) in a woman of an extremely delicate and sensible habit of body, very susceptible of emotions, worn-out by previous diseases or by discharges; when the pulse is, from the beginning, small and contracted; when fainting succeeds more or less rapidly, with convulsions, spasms, pale watery urine, in a word, with all the symptoms of ataxic fever: certainly all idea of peritonitis and gastric inflammation should here be discarded. The latter may, however, (but not always,) be complicated with this state; and ipecacuanha, according to the method of Doulcet, may here be proper, as well as a moderate evacuant, to draw to the stomach and other parts of the animal economy, the vital powers concentrated in the hypogastric region; and it is with these views that I have employed this remedy. We should, at the same time, quickly cover the patient with what are commonly termed *flying* blisters;* the good effects of which I cannot sufficiently praise. The hypogastrium should be covered with flannels embued with spirituous and aromatic liquids; and the strength should be supported by a diet somewhat nourishing. If the bowels are indolent, evacuations should be solicited by emollient and aromatic glysters; but purging should not be induced until the danger is over, and it should be effected with substances at the same time a little tonic, as rhubarb with mucilage of gum arabic. Cinchona, which Franck recommended, has not appeared to me to be useful: I have many times employed it, being deceived by the remissions of the fever, but the paroxysm has always returned with more severity. Musk and camphor may be useful, by inducing an equable diffusion of the vital actions.

But, alas! it is easy to write, and very difficult to execute, and particularly with success. We are often reduced to be no more than spectators of an unequal combat, and to moderate the symptoms. We should endeavour to relieve the vomiting and diarrhœa which frequently occur, and to alleviate the violent pain by glysters of starch and laudanum; and we should favour sweating, if it appears that there is a disposition to relief in this way, by acetate of ammonia, Dover's powder, &c. according to circumstances." (*Journal complément. du Dict. des Sciences Medicales*, Nos. 8 and 9.)

---

* Blistering-plasters applied only for a short time, so as to induce more or less irritation of the skin without vesication. Ed.

